# Somatometric, Training, and Behavioral Profiles of Resistance Training Practitioners and Recreational Exercisers in Greece: A Multivariate Comparative Study

**DOI:** 10.3390/sports14030120

**Published:** 2026-03-19

**Authors:** Ioannis Tsartsapakis, Aglaia Zafeiroudi, Athanasia Chatzipanteli, Maria Gerou

**Affiliations:** 1 Department of Physical Education and Sport Science, Aristotle University of Thessaloniki, 62122 Serres, Greece; miamixan@hotmail.com; 2 Department of Physical Education and Sport Science, University of Thessaly, 42100 Trikala, Greece; azafeiroudi@uth.gr (A.Z.); athxatzipan@pe.uth.gr (A.C.)

**Keywords:** exercise participation, public health, resistance training, recreational exercise, somatometric indicators, training patterns, cluster analysis

## Abstract

This cross-sectional study compared somatometric, training, and behavioral characteristics of adult exercisers in Greece, contrasting self-identified resistance-training practitioners with recreational exercisers. A total of 1187 adults completed a structured questionnaire capturing demographics, self-reported height and weight (BMI), weekly training frequency, session duration, competition participation, and self-reported use of performance-enhancing substances. Given non-normal distributions, analyses used nonparametric tests, binary logistic regression, and two-step cluster analysis based on the elbow method. Resistance-training practitioners reported higher BMI, greater weekly training frequency, and longer session duration than recreational exercisers (all *p* < 0.001). Substance use was more prevalent among resistance-training practitioners and exhibited a marked gender asymmetry, with anabolic-agent use concentrated among men. A logistic regression predicting competition participation identified age, BMI, gender, and education as significant predictors; the model explained a modest proportion of variance (Nagelkerke *R*^2^ = 0.10). Cluster analysis produced four distinct participant profiles differing in BMI, training intensity, and behavioral orientation. These results indicate systematic somatometric and behavioral differences between exercise orientations and demonstrate the utility of multivariate profiling for characterizing heterogeneity in exercise engagement. Findings should be interpreted cautiously because all anthropometric and substance-use measures were self-reported, and BMI cannot distinguish lean from fat mass in resistance-trained populations; future research should prioritize representative sampling and objective somatometric assessment.

## 1. Introduction

Regular participation in exercise is shaped by a complex interplay of demographic, socioeconomic, and somatometric factors that influence both access to physical activity and the intensity of engagement. Variables such as age, education, income, and occupational status interact with measurable indicators including body mass index (BMI), training frequency, and session duration to determine participation patterns and related health outcomes [1,2]. Within the Greek adult population, these factors operate in a context characterized by diverse exercise environments and varying socioeconomic conditions, making it particularly relevant to examine how quantifiable indicators differentiate exercisers across training orientations. While exercise participation is influenced by a range of contextual factors, the present study concentrates on quantifiable variables that can be reliably assessed through structured questionnaires. The analytical focus is placed on quantifiable indicators—BMI, training behavior, education, income, and substance use—which allow for empirical comparison across exerciser groups [3]. These variables were selected because they represent standardized and widely used indicators in exercise science research, enabling consistent measurement across diverse exercise orientations and allowing for direct comparison within the Greek adult population. Their inclusion reflects both their established relevance in previous empirical studies and the methodological feasibility of assessing them reliably through structured questionnaires. This empirically oriented approach is adopted to describe how measurable variables cluster across exercise orientations, a methodologically appropriate choice given the exploratory nature of the study, rather than to develop a sociological theory of physical activity.

Accordingly, the study’s primary analytical focus is the comparison between resistance-training practitioners and recreational exercisers, with all supplementary analyses serving to enhance the interpretation of this central contrast.

### 1.1. Exercise Participation as a Social and Health-Related Practice

Physical activity is consistently associated with reduced cardiometabolic risk, improved psychological functioning, and enhanced quality of life [4,5,6,7]. Indicators such as BMI, training frequency, and substance use serve as measurable proxies for behavioral orientation and public health risk, as they reflect common patterns of exercise engagement observed within the Greek adult population. Excessive training and the use of pharmacological enhancement substances have been linked to adverse metabolic, cardiovascular, and psychiatric outcomes [8,9], underscoring the importance of examining exercise participation through a somatometric and behavioral lens. This approach aligns with the present study’s focus on quantifiable indicators rather than sociocultural interpretations.

Socioeconomic status is associated with differences in exercise participation. Individuals with higher socioeconomic resources often report greater access to structured exercise opportunities, more stable engagement patterns, and increased likelihood of participating in organized fitness activities [10]. Recent evidence suggests that socioeconomic factors may also be related to preferences for specific exercise modalities, including resistance-based training, where economic status and contextual factors contribute to differences in participation and body-image orientation [2,11,12,13]. In this study, socioeconomic status is operationalized through education and income, reflecting its most commonly used measurable components in exercise science research. Within the Greek context, however, multivariate comparisons across exercise types remain limited, a point reflected in the scarcity of studies integrating demographic and somatometric variables within a single analytical framework, highlighting the need for empirical analyses that integrate demographic and somatometric variables.

### 1.2. Resistance Training as a High-Intensity, Performance-Oriented Modality

Resistance-training practitioners typically engage in structured programs aimed at muscular development, strength improvement, and esthetic enhancement [12]. In this study, the term refers to individuals who consistently perform resistance-based training with performance- or physique-oriented goals, without implying professional athletic status. Prior research shows that resistance-focused training is associated with higher training intensity, stricter routines, and greater prevalence of performance-enhancing substance use compared to recreational exercise [14,15]. Pharmacological enhancement practices, including anabolic-androgenic steroids, have been linked to cardiovascular strain, endocrine disruption, and psychiatric effects [8,16]. Beyond anabolic agents, resistance-training settings may also involve other appearance- or performance-oriented practices, although the present study assessed only self-reported use of performance-enhancing substances. In the present study, substance use was operationalized through self-reported use of performance-enhancing agents, aligning the measured variables with the behaviors described in the literature. These behaviors reflect complex motivational and behavioral patterns among amateur practitioners, as highlighted in recent systematic evidence [17].

Although resistance training has been examined through subcultural and symbolic frameworks [14,18,19], these perspectives are acknowledged only as background, as the present study focuses on measurable somatometric and behavioral indicators. The emphasis remains on quantifiable variables, BMI, training frequency, duration, education, income, and substance use, that differentiate exercisers in empirically assessable ways and align with the study’s comparative design.

### 1.3. Recreational Exercise as a Health-Promoting Activity

Recreational exercise contributes to physical health, psychological well-being, and social integration [2,5,20]. It emphasizes enjoyment, stress management, and personal balance, attracting individuals across socioeconomic backgrounds. Empirical evidence links recreational activity with stable BMI values, moderate training frequency, and sustainable exercise duration, while regular participation reduces the risk of chronic disease and supports cardiometabolic health [2,5]. In this study, recreational exercisers are defined as individuals who engage in fitness or sport activities regularly without competitive or esthetic goals. Recreational exercise included a range of non-competitive physical activities (e.g., fitness programs, recreational sports, aerobic activities), but specific activity types were not used as analytical variables.

Socioeconomic indicators such as education and income influence recreational engagement [2,21], while psychological benefits, including reduced anxiety and improved quality of life, reinforce participation [22,23]. Despite these findings, multivariate comparisons between recreational exercisers and resistance-training practitioners remain limited in the Greek population, underscoring the need for empirical analyses that integrate demographic and somatometric characteristics. This subsection therefore establishes the relevance of recreational exercise as a distinct behavioral orientation that can be empirically contrasted with resistance training within the present study.

### 1.4. Substance Use, BMI, and Competitive Participation in Resistance Training

Substance use, body composition, and competitive engagement are closely interconnected in resistance-training populations. Anabolic-androgenic steroid use is prevalent among non-professional athletes, driven by performance enhancement, esthetic goals, and peer influence [8,24,25].

BMI is frequently used as a proxy for body composition, though its limitations in athletic populations are well documented. Research indicates that BMI and body-image dissatisfaction influence exercise motivation and participation patterns [26,27]. Elevated BMI in resistance-training contexts may reflect increased lean mass, yet higher BMI values, regardless of composition, are associated with long-term cardiometabolic risks [28]. In the present analysis, BMI was interpreted descriptively without additional sensitivity checks, acknowledging these limitations in resistance-trained individuals. Regional studies report substantial prevalence of steroid use among resistance-training athletes in European and Mediterranean populations [29,30], while comparative research highlights differences between natural and enhanced athletes in training rigidity and stress levels [31]. These findings underscore the importance of examining substance use, BMI, and training behavior as interconnected indicators of risk within resistance-training settings [32,33].

The central analytical aim of the present study is to compare resistance-training practitioners and recreational exercisers using standardized somatometric and behavioral indicators. All additional analyses are exploratory and serve to contextualize this primary comparison.

### 1.5. Study Rationale and Research Hypotheses

Despite extensive international research, the Greek population remains underrepresented in studies that integrate demographic, somatometric, and behavioral variables across exercise types and gender. Existing studies have examined isolated aspects such as body image dissatisfaction, physique anxiety, or dietary adherence, but not within a multivariate framework that accounts for clustering of behaviors and competitive engagement. The present study addresses this gap by employing a comparative and multivariate design to analyze somatometric and behavioral profiles of resistance training practitioners and recreational exercisers in Greece. This approach allows for the identification of distinct patterns of exercise engagement and contributes to a more comprehensive understanding of how demographic and physical variables shape participation.

Based on prior literature, six exploratory hypotheses were formulated. Each hypothesis corresponds directly to variables measured in the study, ensuring alignment between the conceptual aims and the operational indicators. These hypotheses are not intended to test a theoretical model but to guide the empirical examination of measurable somatometric and behavioral indicators within a unified analytical framework. An exploratory approach was preferred because no established theoretical model integrates these specific variables within the Greek exercise context. Their purpose is descriptive and comparative, reflecting the study’s focus on quantifiable variables rather than sociological constructs.

1. Resistance training practitioners will report higher training frequency and session duration than recreational exercisers.
2. Lower education and income will be associated with higher BMI and weight-related challenges.
3. Individuals with reduced socioeconomic resources will engage more frequently in resistance-oriented training.
4. Substance use will be more prevalent among resistance training practitioners and will correlate with BMI, with gender-specific variation.
5. Competition participation will be predicted by age, BMI, exercise type, and gender.
6. Distinct exerciser profiles will emerge from somatometric and behavioral variables, reflecting the diversity of fitness engagement in Greece.

These hypotheses are exploratory and serve to structure the empirical analysis rather than to propose or validate a theoretical framework. The study aims to describe patterns of exercise engagement using standardized variables and validated instruments, emphasizing measurable differentiation within sports and exercise science. Together, these hypotheses provide a coherent exploratory framework that aligns directly with the measurable variables included in the study.

## 2. Materials and Methods

### 2.1. Participants

A total of 1248 questionnaires were initially collected. Following the exclusion of 61 cases due to incomplete data or failure to meet the inclusion criteria regarding training experience and frequency, the final sample comprised 1187 adult exercisers aged 18 to 59 years (*M* = 28.82, *SD* = 7.19). This represents an exclusion rate of 4.9%. Cases were excluded if they failed to meet the minimum thresholds for training duration and frequency or provided incomplete demographic or anthropometric information. Participants were recruited through convenience sampling from fitness and recreational facilities across Greece. Recruitment took place in publicly accessible private gyms and recreational centers located in urban and semi-urban areas. Elite or specialized training centers were not included, ensuring that the sample reflected typical exercise environments accessible to the general population. Convenience sampling was chosen for feasibility, although it does not yield a nationally representative sample; thus, the generalizability of the findings is limited, and external validity should be interpreted cautiously.

Recruitment sites included facilities in Northern Greece (Thessaloniki and Serres), Central Greece (Trikala and Larissa), and the wider Athens metropolitan area, providing diversity in regional representation. Inclusion criteria required participants to have at least two years of continuous training experience and to engage in a minimum of three weekly sessions lasting at least 45 min. The eligibility criteria were based exclusively on self-reported training history and were not independently verified. To ensure consistent and stable exercise behavior, the thresholds were selected to minimize variability associated with beginners or irregular participation.

Participants were classified into four groups based on self-reported training orientation and gender: male resistance-training practitioners (*n* = 363), female resistance-training practitioners (*n* = 183), male recreational exercisers (*n* = 343), and female recreational exercisers (*n* = 298). Resistance-training practitioners were defined as individuals who regularly engaged in structured resistance-based programs oriented toward strength, hypertrophy, or physique development, regardless of competitive status. This category included recreational lifters, hypertrophy-oriented lifters, aspiring competitors, and non-competitive advanced lifters whose primary mode of training involved resistance exercise. Participants assigned to this group also reported that they had not engaged in any other structured activity (e.g., yoga, Pilates, functional training, running, cycling, swimming) during the previous two years, ensuring exclusive involvement in resistance-based exercise.

Recreational exercisers were defined as individuals participating in structured physical activities performed regularly without competitive or esthetic goals. This category encompassed fitness programs, non-competitive team sports, running, cycling, swimming, climbing, dynamic walking, dance, and other forms of exercise undertaken for health and wellness. Participants classified as recreational exercisers reported that they had not engaged in gym-based resistance training during the previous two years, ensuring clear separation between groups and eliminating the presence of mixed or hybrid exercisers in the dataset. Activities were classified as recreational only when practiced outside formal sport organizations or competitive contexts, ensuring consistency in group assignment.

Somatometric indicators, including body mass index (BMI), were incorporated to enable comparisons across groups and to serve as proxies for general health-related risk. BMI is a widely established index of relative weight and obesity [34]. Nevertheless, BMI presents well-documented limitations in resistance-training populations, since elevated lean mass may increase BMI values without indicating excess adiposity. No alternative somatometric indicators were included, as the study relied exclusively on self-reported measures; this constraint was acknowledged when interpreting intergroup differences [26,27]. All participants completed the survey voluntarily, provided informed consent, and their data were processed anonymously. Anthropometric and substance-use variables were self-reported, with no external validation or objective verification procedures implemented. This reliance on self-report should be considered when interpreting somatometric and behavioral differences across groups.

The classification of participants into resistance-training practitioners and recreational exercisers was based strictly on self-reported training orientation and gender, reflecting observable exercise behaviors rather than sociological categories. This operational definition ensured clarity in group comparisons and alignment with the empirical scope of the study.

Descriptive statistics for age, height, weight, and BMI across groups are presented in Table 1.

### 2.2. Procedure

Participants completed a structured questionnaire assessing demographic variables (age, marital status, employment, education, income), anthropometric measures (self-reported height, weight, and weight-related concerns), and exercise-related variables (exercise type, weekly frequency, daily duration, and double-session training). BMI was calculated using the standard formula (kg/m^2^). Weight-related categories were operationalized according to self-reported BMI ranges and questionnaire items addressing weight-related concerns, ensuring clarity and consistency in variable interpretation.

To ensure procedural transparency, the data-collection process followed a linear sequence consisting of six stages. Participants were first recruited from fitness and recreational facilities across Greece. Inclusion criteria were then applied to confirm eligibility based on training experience and weekly exercise frequency. Eligible individuals were subsequently classified into four groups according to self-reported training orientation and gender. Participants then completed the structured questionnaire, after which all responses were screened and processed for completeness and internal consistency. Finally, the dataset was prepared for statistical analysis using nonparametric tests, logistic regression, and cluster analysis.

In addition to the custom questionnaire, the Muscle Dysmorphia Inventory (MDI) developed by Rhea et al. [35] is a 27-item instrument based on the diagnostic criteria proposed by Lantz et al. [36]. Items are rated on a six-point Likert scale ranging from “never” to “always” and assess six independent dimensions: size and symmetry concerns, body exposure protection, exercise dependence, supplement use, dietary behavior, and pharmacological use. The instrument was originally validated in three separate studies, demonstrating reliable assessment of psychological characteristics related to muscle dysmorphia. The MDI is not intended as a diagnostic tool and has not been applied to adolescent populations, a limitation noted in its original validation [35].

The questionnaire was first introduced in the Greek context through a postgraduate thesis at Aristotle University of Thessaloniki [37], where linguistic translation, cultural adjustment, and reliability testing were conducted. The thesis is available in the institutional repository of AUTH (https://ikee.lib.auth.gr/record/128559/files/GRI-2012-8078.pdf, accessed on 18 December 2025). Subsequent studies have employed this Greek version in non-clinical exercise populations, confirming its practical applicability [38,39]. In the present study, the MDI was used solely to assess behavioral tendencies related to muscle dysmorphia, with emphasis on the pharmacological-use dimension, which demonstrated satisfactory internal consistency (Cronbach’s α = 0.82).

### 2.3. Data Analysis

Statistical analyses were conducted using IBM SPSS Statistics, version 29.0 (IBM Corp., Armonk, NY, USA). The dataset was examined for completeness and internal consistency, with no missing values retained. Descriptive statistics were calculated for all variables. For continuous variables, means and standard deviations were computed, while categorical variables were summarized using frequency distributions and percentages.

Nonparametric methods were employed due to the ordinal level of several variables and the non-normal distribution of continuous measures. Normality was examined using Shapiro–Wilk tests, which supported the use of nonparametric procedures. Mann–Whitney U tests were used to compare training frequency, training duration, BMI, and weight-related history across groups. Kruskal–Wallis and Chi-square tests examined associations between competition participation and demographic variables. Binary logistic regression was used to predict competition participation from age, BMI, income, education, and exercise type. Model reliability was assessed using Hosmer–Lemeshow goodness of fit tests. Effect sizes were reported for all relevant analyses to support interpretation of group differences.

Cluster analysis was conducted using a Two-Step procedure to identify latent profiles based on age, BMI, training behavior, and income. This method accommodates mixed data types and is suitable for exploratory segmentation. Continuous variables were normalized using min -max scaling, and categorical variables were dummy coded. The optimal number of clusters was determined using the elbow method, which was preferred over BIC because it provided clearer visual identification of the point of diminishing returns in within-cluster variance for this dataset. Preliminary BIC values did not show a clear inflection point, whereas the elbow criterion yielded a more stable and interpretable solution for this dataset. Post hoc comparisons were performed using Kruskal–Wallis and Chi-square tests to examine differences across clusters. Statistical significance was set at *p* < 0.05.

### 2.4. Ethical Considerations

All procedures were conducted in accordance with the Declaration of Helsinki. Participation was voluntary, informed consent was obtained from all individuals prior to data collection, and no identifying information was collected. All responses were processed anonymously. The study protocol was approved by the Ethics Committee of the Department of Physical Education and Sport Science, University of Thessaly (Protocol No. 2252, 3/2/11 October 2023). No incentives were provided, and participants retained the right to withdraw at any stage without consequences.

## 3. Results

### 3.1. Descriptive Analysis

Descriptive statistics were calculated for all continuous variables across the four subgroups defined by gender and training orientation. Resistance training practitioners (RTP) demonstrated consistently higher somatometric and training-related values than recreational exercisers, with male RTP participants showing the highest overall levels. Female RTP participants also exceeded both recreational groups across all indicators.

BMI was interpreted with caution, as elevated values among resistance-trained individuals may reflect increased lean mass rather than adiposity. Interpretation was applied uniformly across male and female RTP groups, as no sex-specific sensitivity analyses were conducted. In this study, BMI served as a practical somatometric indicator, with interpretations contextualized according to training orientation. BMI differences should be interpreted cautiously, as higher values in resistance-trained individuals may reflect lean mass rather than adiposity, and no sensitivity analyses were conducted.

Age distribution was similar across subgroups, with mean values around 28–29 years. Group differences in BMI and training frequency were statistically significant (Cohen’s *d* = 0.52 for BMI; *d* = 0.47 for training frequency), as shown in Table 2, which reports the exact test statistics and effect sizes.

### 3.2. Comparative Frequency Analysis of Sociodemographic and Behavioral Variables

Frequency analyses examined sociodemographic and behavioral characteristics across the four groups. Effect sizes were calculated for all key group comparisons to support interpretive clarity and are reported alongside the corresponding test statistics in the tables. Group differences reflect raw, unadjusted comparisons, as no covariate controls (e.g., age or income) were applied. Substance-use percentages are reported in Table 3 to avoid redundancy in the text. As shown in Table 3, resistance-training practitioners, particularly males, demonstrated the highest training intensity and the most frequent use of enhancement substances, whereas female recreational exercisers showed minimal engagement in competitive or pharmacological behaviors.

Educational attainment was generally high across the sample, with recreational male participants displaying a more even distribution across education levels. Income patterns varied substantially between groups, reflecting the composition of the convenience sample, especially among female RTP participants, whose income levels were influenced by the presence of students and early-career individuals.

Differences in substance use between male RTP participants and all other groups were statistically significant and large in magnitude (Cramer’s *V* = 0.41). Competition participation was also more common among male RTP participants, although the effect size was small (Cramer’s *V* = 0.12).

### 3.3. Assessment of Distributional Assumptions

Normality tests (Kolmogorov–Smirnov) indicated significant deviations from normality for all continuous variables (*p* < 0.001). Due to the consistent violation of distributional assumptions, nonparametric methods were employed throughout the analysis to ensure robustness.

### 3.4. Reliability Analysis

The pharmacological-use dimension of the MDI demonstrated satisfactory internal consistency (Cronbach’s α = 0.87), consistent with the original validation study [35], supporting its use in the behavioral analyses.

### 3.5. Evaluation of Hypothesis 1: Differences in Training Frequency and Duration Between Bodybuilders and Recreational Exercisers

#### 3.5.1. Mann–Whitney U Test: Overall Comparison

Resistance training practitioners trained significantly more frequently and for longer durations than recreational exercisers, with large and small-to-medium effect sizes, respectively (all *p* < 0.001).

#### 3.5.2. Mann–Whitney U Test: Gender-Specific Comparisons

Among males, RTP participants showed substantially higher training frequency and longer session duration than recreational exercisers, with large and medium effect sizes. Among females, RTP participants trained more frequently, but session duration did not differ significantly.

#### 3.5.3. Kruskal–Wallis Test: Four-Group Comparison

Training frequency and duration differed significantly across all four subgroups, with RTP participants ranking highest on both indicators. Table 4 presents the mean ranks for each group.

Overall, Hypothesis 1 was supported. Resistance training practitioners demonstrated higher training engagement than recreational exercisers, with gender moderating the magnitude of these differences.

### 3.6. Socioeconomic Status and Somatic Regulation: Associations Between Income, Education, BMI, and Weight History

This hypothesis examined whether socioeconomic status was associated with BMI and weight-related difficulties.

#### 3.6.1. Correlations Between Socioeconomic Indicators and BMI

Income showed a significant negative association with BMI (ρ = −0.256, *p* < 0.001, R^2^ = 0.07), indicating higher BMI among lower-income individuals. No significant relationship emerged between education and BMI (ρ = 0.024, *p* = 0.414), suggesting that education did not independently predict body composition in this sample.

#### 3.6.2. BMI Differences Across Income Groups

BMI differed significantly across income categories, *H*(4) = 108.598, *p* < 0.001, η^2^ = 0.09. As shown in Table 5, lower-income groups exhibited higher BMI values and a greater prevalence of weight-related difficulties, whereas higher-income groups reported lower BMI and fewer difficulties.

#### 3.6.3. Educational Attainment Across Income Groups

Educational attainment differed significantly across income groups, *H*(4) = 155.8, *p* < 0.001, η^2^ = 0.12, with higher income levels corresponding to higher educational attainment.

#### 3.6.4. Weight-Related Difficulties and Socioeconomic Status

Income was significantly associated with weight-related difficulties, χ^2^(4) = 16.8, *p* = 0.002, Cramer’s *V* = 0.12, with lower-income individuals more likely to report challenges in weight regulation. Education was not significantly related to weight-related difficulties, χ^2^(2) = 4.96, *p* = 0.084.

### 3.7. Socioeconomic Predictors of Exercise Type: Income and Education as Determinants of Bodybuilding Orientation

This hypothesis examined whether lower income and educational attainment predict engagement in bodybuilding-oriented training compared to recreational exercise.

#### 3.7.1. Crosstab Analysis

Resistance-training participation was more common among lower-income individuals, although the association did not reach statistical significance, χ^2^(4) = 8.97, *p* = 0.062, Cramer’s *V* = 0.08.

Educational attainment showed a stronger and statistically significant association with exercise type, χ^2^(2) = 49.8, *p* < 0.001, Cramer’s *V* = 0.20, with individuals of lower educational levels more likely to engage in resistance training.

#### 3.7.2. Logistic Regression Analysis

Binary logistic regression examined whether income and education predicted exercise type. The overall model was statistically significant, χ^2^(6) = 64.4, *p* < 0.001, Nagelkerke *R*^2^ = 0.071, indicating modest explanatory power. Education emerged as the strongest predictor: individuals with postgraduate education were significantly less likely to engage in resistance training compared to those with primary or secondary education (B = 1.330, *p* < 0.001, Exp(B) = 3.782). Income effects were weaker, with only the €1001–1500 category approaching significance (B = −0.599, *p* = 0.054). Table 6 presents the full regression coefficients.

In summary, the hypothesis was partially supported: education showed a clear association with exercise type, whereas income demonstrated limited predictive value.

### 3.8. Enhancement Practices and Somatic Regulation: Substance Use, BMI, and Gender-Specific Patterns

This hypothesis examined whether substance use was more frequent among resistance-training practitioners and whether it related to BMI, with attention to gender differences. Significant associations emerged across all three substance categories. For steroids and diuretics, frequent and occasional use was concentrated among RTP participants (all *p* < 0.001). Laxative use showed a mixed pattern, with occasional use more common among RTP participants and frequent use more common among recreational exercisers (*p* < 0.001).

Gender comparisons indicated that frequent use of steroids, laxatives, and diuretics occurred almost exclusively among men, while female participants reported negligible use. All chi-square tests were significant (*p* < 0.001).

A Mann–Whitney U test showed that steroid users had significantly higher BMI than non-users (*U* = 26481.000, *p* < 0.001), a pattern that remained significant among men. No female participants reported steroid use. Table 7 and Table 8 summarize substance-use patterns.

Overall, findings supported the hypothesis, indicating that substance use was concentrated among male resistance trainees and associated with elevated BMI.

### 3.9. Predictors of Competition Participation: Age, BMI, Exercise Type, and Gender

A binary logistic regression assessed whether age, BMI, exercise type, and gender predicted competition participation. The model was statistically significant, χ^2^(4) = 40.484, *p* < 0.001, Nagelkerke *R*^2^ = 0.100, with acceptable fit (Hosmer–Lemeshow *p* = 0.070). Classification accuracy reflected the low prevalence of competition participation.

Age and BMI were significant positive predictors: older participants and those with higher BMI were more likely to compete. Gender was also significant, with female participants less likely to report competition involvement. Exercise type did not reach significance (*p* = 0.182). Table 9 presents the regression coefficients.

In summary, the hypothesis was partially supported. Age, BMI, and gender predicted competition participation, whereas exercise type did not exert an independent effect.

### 3.10. Clustered Profiles of Exercisers Based on Somatometric and Behavioral Variables

The sixth hypothesis proposed that distinct exerciser profiles would emerge from somatometric and behavioral variables. A two-step cluster analysis using standardized somatometric indicators and categorical variables (exercise type, competition participation, substance use) identified four clusters among 1187 valid cases, indicating differentiated patterns in training behavior and body composition.

Cluster 1 included individuals with moderate BMI and low training frequency, primarily recreational exercisers. Cluster 2 showed higher training frequency and duration, a greater proportion of bodybuilding participants, and elevated BMI. Cluster 3 comprised individuals with lower BMI and minimal substance use across mixed exercise types. Cluster 4 was characterized by higher BMI, frequent training, and greater representation of competition-oriented participants.

A one-way ANOVA on standardized scores confirmed significant differences across clusters for BMI (*F*_(3,1183)_ = 42.7, *p* < 0.001, η^2^ = 0.10), training frequency (*F*_(3,1183)_ = 51.2, *p* < 0.001, η^2^ = 0.12), and training duration (*F*_(3,1183)_ = 38.5, *p* < 0.001, η^2^ = 0.09), indicating medium effect sizes and supporting the hypothesis.

**Figure 1:**
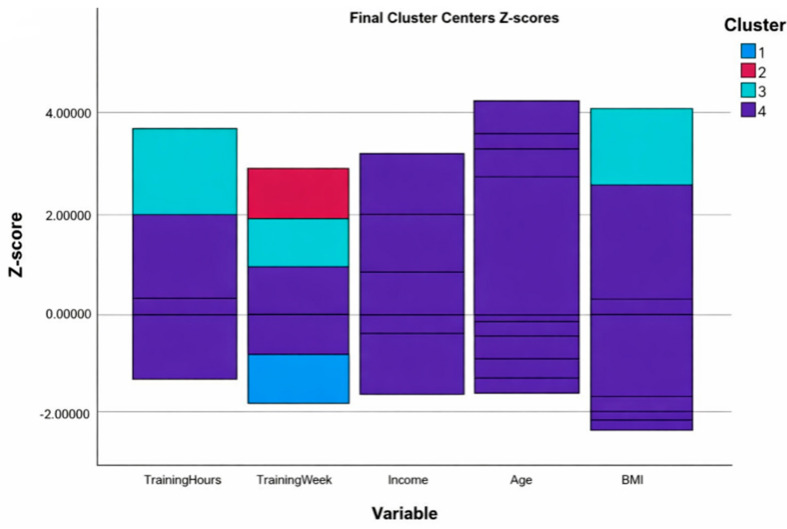
Final Cluster Centers (Z-scores) Across Somatometric and Behavioral Variables. Note. Z-scores represent standardized values for each variable within the identified clusters. Positive scores indicate above-average values relative to the sample mean, while negative scores reflect below-average levels.

To support interpretation, original units and variable ranges are reported in Table 2 and Table 3. The cluster centers presented in Figure 1 are standardized (Z-scores), allowing for visual comparison across variables with different scales. This representation is descriptive and not intended for predictive or diagnostic use.

Figure 1 presents the standardized cluster centers for training duration, training frequency, income, age, and BMI. Cluster 2 displayed elevated scores across training-related and somatometric dimensions, Cluster 1 showed moderate values, and Clusters 3 and 4 exhibited lower scores across most variables, confirming the segmentation identified in the analysis.

## 4. Discussion

The present study examined how demographic, somatometric, and behavioral variables influence exercise engagement among adult resistance-training practitioners and recreational exercisers in Greece. By integrating indicators such as training frequency, training duration, BMI, income, educational attainment, and substance use into a multivariate analytical framework, the study identified differentiated patterns of participation and physiological investment. The comparative design and the application of cluster analysis enabled the detection of latent profiles based on combined behavioral and physical characteristics, offering a more comprehensive understanding of how individual and socioeconomic factors shape exercise behavior in contemporary fitness environments.

The first hypothesis proposed that resistance training practitioners would engage in more frequent and longer training sessions than recreational exercisers. The findings confirmed this differentiation, with male practitioners showing the highest values and female practitioners also exceeding recreational exercisers. Training frequency and session duration showed the strongest between-group differences, aligning with research indicating that structured resistance training is associated with higher weekly volumes and more specialized goals [40,41]. Extended training duration may reflect achievement-oriented motivation and adherence to hypertrophy-focused routines, although excessively long sessions may compromise training efficiency. Excessive exercise has been linked to dependence symptoms and muscle dysmorphia tendencies, particularly among individuals pursuing physique-oriented goals [30,42]. These findings confirm behavioral divergence between resistance-training practitioners and recreational exercisers, consistent with literature showing that resistance-training-oriented individuals often pursue externally regulated goals, whereas recreational exercisers prioritize intrinsic motives and general health maintenance [29,43].

The second hypothesis proposed that lower educational attainment and income would be associated with higher BMI and increased prevalence of weight-related difficulties. The findings partially confirmed this hypothesis. Income was associated with both body composition and reported difficulties in weight regulation, with lower-income individuals exhibiting higher BMI values and more frequent challenges. This pattern aligns with research linking economic disadvantage to elevated obesity risk, reduced access to structured exercise environments, and limited availability of nutritionally adequate diets [44,45]. In contrast, educational attainment did not independently predict BMI or weight-related difficulties. This divergence may reflect factors not captured in the present analysis, such as variability in access to facilities, differences in health literacy, or disparities in nutritional practices [46,47]. The strongest somatometric divergence was observed in BMI, which differed consistently across income levels. Elevated BMI, even when reflecting lean mass, has been linked to long-term cardiometabolic risk [32], while substance-use behaviors documented among resistance-trained individuals are associated with adverse cardiovascular and psychiatric outcomes [9,48]. These findings highlight the public health relevance of economic stratification in exercise engagement.

Hypothesis 3 proposed that male participants would report higher engagement in enhancement practices, including supplement use and performance-oriented behaviors. The findings supported this hypothesis, revealing a clear gendered pattern in enhancement strategies. Male participants reported significantly higher prevalence of supplement use and pharmacological enhancement, consistent with prior studies [30,42]. Educational attainment also predicted exercise orientation, with lower educational levels associated with resistance-training-related practices and higher levels associated with reduced enhancement behaviors [44,45]. These results remain exploratory and hypothesis-generating, based on measured variables of BMI, exercise type, substance use, income, and education. The strongest behavioral divergence was observed in substance-use patterns, which were concentrated among male resistance-training practitioners. The health implications are notable, as male-dominated enhancement practices have been associated with increased cardiovascular and psychiatric risk [8].

Hypothesis 4 examined whether substance use is more prevalent among resistance-training practitioners and whether it correlates with BMI, with attention to gender-specific patterns. The findings strongly supported this hypothesis, revealing consistent associations between exercise type and substance use. Anabolic agents were reported exclusively by resistance-training practitioners, while non-use was more common among recreational exercisers. Similar trends were observed for diuretics, predominantly used by resistance-training-oriented individuals. Laxative use showed a more complex distribution, with occasional use among resistance-training practitioners and more frequent use among recreational exercisers. These results confirm differentiated substance-use strategies across exercise modalities, consistent with research documenting higher prevalence of pharmacological enhancement among individuals engaged in structured resistance training [8,49,50].

Gender comparisons revealed a pronounced asymmetry. All frequent users of enhancement substances were male, while female participants reported minimal use. Gender asymmetry may partly reflect reporting bias, as social-desirability pressures could lead to underreporting among female participants. Evidence also indicates that men are more likely to engage in performance-oriented enhancement practices, including anabolic-androgenic steroid use [8,49]. The association between substance use and BMI further reinforces the hypothesis. Users of anabolic agents tended to exhibit higher BMI values, likely reflecting increased lean mass rather than adiposity, and no objective body-composition assessments were available. This interpretation aligns with research showing that anabolic steroid use among recreational resistance-trained individuals is associated with increased muscle mass, altered lipid profiles, elevated blood pressure, and changes in cardiac structure [50,51,52]. The use of diuretics and laxatives further supports this interpretation, as these substances are commonly employed to manipulate body water and achieve a leaner appearance, particularly in preparation for physique-oriented events.

Misuse of anabolic agents, laxatives, and diuretics is not confined to professional athletes but is also observed among amateurs [53], often without medical consultation [54]. Such practices can have serious health consequences, including electrolyte imbalance, cardiac strain, and psychological disturbances. Beyond physiological risks, reliance on these substances raises ethical concerns, as it promotes unhealthy and potentially dangerous behaviors [54]. These findings highlight the broader public health implications of substance-use patterns in resistance-training contexts.

Hypothesis 5 examined whether competition participation could be predicted by age, BMI, exercise type, and gender. The findings partially supported this hypothesis. Age and BMI emerged as significant predictors of competitive involvement, aligning with evidence indicating that cumulative training exposure and physiological investment contribute to competition readiness [21,23]. Individuals with higher BMI values were more likely to report competition experience, a pattern that in this sample likely reflects greater lean mass and muscular development rather than adiposity.

Gender also influenced competition participation, with female participants being less likely to report involvement, consistent with research documenting gender disparities in sport participation [55,56,57]. Exercise type did not independently predict competition participation once age and BMI were controlled [58], illustrating the overlap between interrelated predictors and supporting the view that competitive engagement is shaped more by long-term physical investment and self-determined goal pursuit than by categorical exercise orientation [59,60].

Collectively, the findings indicate that age, BMI, and gender were the strongest predictors of competition participation, whereas exercise type did not provide additional explanatory value. Elevated BMI, even when reflecting lean mass, has been linked to long-term cardiometabolic risk [28], underscoring the need for preventive monitoring. However, the logistic regression model showed low Nagelkerke *R*^2^ values, indicating limited explanatory power; therefore, the findings should be interpreted descriptively rather than as a predictive model of competition participation.

Hypothesis 6 proposed that distinct exerciser profiles would emerge based on somatometric and behavioral variables. The findings supported this hypothesis, revealing four differentiated clusters representing meaningful segmentation in training behavior and body composition. Each cluster reflected a specific configuration of BMI, training frequency, training duration, exercise type, and substance use, indicating that measurable somatometric and behavioral indicators can differentiate exerciser subgroups. In summary, the four clusters reflected moderate BMI with low training frequency, high-frequency and high-duration resistance-training engagement with elevated BMI, low BMI with minimal substance use, and high BMI with frequent training and greater competitive involvement.

One group included individuals with moderate BMI and low training frequency, primarily engaged in recreational exercise. Another was characterized by high training frequency and duration, elevated BMI, and a greater proportion of resistance-training practitioners, suggesting a more intensive training orientation. A third cluster comprised individuals with lower BMI and minimal substance use, reflecting a more balanced or health-oriented engagement. The final group included participants with high BMI and frequent training, with notable representation of competition-oriented individuals, indicating elevated physiological investment and performance-driven behavior.

The visual representation of cluster centers reinforced these distinctions. Clusters with elevated values in training-related and somatometric dimensions indicated higher levels of commitment and physiological adaptation, while others showed reduced intensity and lower body-composition indices. These patterns align with research demonstrating that clustering techniques can differentiate exerciser profiles based on behavioral and physiological variables [24,61]. Income did not emerge as a dominant differentiator across clusters, suggesting that behavioral and somatometric variables may exert greater influence on profile formation than socioeconomic status alone. While Figure 1 offers a descriptive visualization of cluster differences, its interpretation should remain exploratory, as no external validation or stability indices were applied.

The clustering structure provides exploratory evidence of heterogeneity in fitness engagement. The emergence of distinct clusters illustrates systematic variation in training behavior and somatometric status, supporting the hypothesis and demonstrating that exercisers in Greece represent diverse profiles shaped by differences in training intensity, body composition, exercise orientation, and enhancement practices. These results remain exploratory and hypothesis-generating, based on self-reported measures and preliminary segmentation. Future research should employ representative samples, validated scales, and advanced clustering techniques such as latent class analysis to strengthen the reliability and generalizability of these findings [62]. Clustering approaches have been increasingly applied in public health research to identify risk groups and inform preventive strategies, particularly in relation to somatic risk, behavioral patterns, and health-related outcomes [31,63].

The findings highlight the central role of body composition, training intensity, and socioeconomic status in shaping exercise engagement. They contribute to the broader literature by validating established associations and identifying emerging behavioral configurations. The inclusion of both sexes allowed for the identification of gender-specific patterns in training engagement and enhancement practices. Overall, the study underscores the importance of demographic and socioeconomic variation when examining exercise participation and supports multidimensional approaches grounded in measurable somatometric and behavioral indicators rather than sociological constructs.

### Limitations

Several methodological limitations must be acknowledged. First, the use of convenience sampling restricts generalizability, as participants were recruited from fitness and recreational facilities across Greece, potentially overrepresenting individuals with stable access to structured exercise environments. This may have influenced age distribution, gender balance, and regional representation, limiting external validity. Second, reliance on self-reported data introduces biases related to social desirability and recall accuracy, particularly for sensitive items such as pharmacological enhancement. Self-reported height, weight, and substance use are especially vulnerable to misreporting, and underreporting may be more pronounced among female participants. Third, the cross-sectional design limits causal inference, since associations between BMI, training frequency, and substance use cannot establish directionality or temporal ordering. Thus, observed associations between socioeconomic status, BMI, and exercise type should be interpreted descriptively rather than causally. Fourth, the operational definition of exercise type was based on self-categorization, which may not fully capture the complexity of training orientation or hybrid practices. Fifth, although the cluster analysis revealed meaningful segmentation, its explanatory power was modest and relied primarily on descriptive patterns rather than inferential modeling. Finally, gender comparisons were constrained by the uneven distribution of enhancement practices, underscoring the need for gender-sensitive instruments and qualitative methodologies that can more accurately capture differences in disclosure, motivation, and behavioral expression.

## 5. Conclusions

This study investigated the somatometric and behavioral characteristics of adult exercisers in Greece, comparing resistance-training-oriented and recreational participants across gender. The analysis incorporated BMI, exercise type, training frequency and duration, substance use, and competition participation, aiming to identify systematic patterns of engagement.

The findings revealed differentiated profiles of exercisers. Resistance-training practitioners reported higher training involvement, elevated BMI values, and greater prevalence of enhancement practices, particularly among men. Competition participation was influenced by age, BMI, and gender, whereas exercise type did not independently predict involvement. Cluster analysis identified four preliminary profiles, offering insight into heterogeneity in training behavior and body composition. Taken together, these results indicate that exercise orientation is associated with consistent somatometric and behavioral patterns, reflecting broader differences in training intensity, body-related attitudes, and engagement practices.

These conclusions should be interpreted with caution due to several methodological constraints, including reliance on convenience sampling, self-reported anthropometric and behavioral data, potential underreporting of substance use, and the limited explanatory power of predictive models. BMI was used as a proxy for body composition, although its limitations in resistance-trained populations are well documented. Given the exploratory design and sampling approach, the findings should be viewed as indicative rather than definitive.

Overall, the study provides an initial mapping of exerciser profiles in Greece, highlighting both the potential risks associated with intensive resistance-training practices and the comparatively stable patterns observed among recreational participants. These findings may inform practical applications, such as targeted screening for enhancement-related behaviors or tailoring training guidance according to exercise orientation. Future research using representative samples, objective somatometric assessments, and validated behavioral measures is needed to substantiate and extend these findings. Objective assessments such as body-composition analysis or basic strength testing would strengthen future evaluations without altering the conceptual scope of similar studies.

## Figures and Tables

**Table 1:** Somatometric characteristics of participants (N = 1187).

Variable	Total Sample (*N* = 1187) (*M ± SD*)	Male RTP (*n* = 363) (*M ± SD*)	Female RTP (*n* = 183) (*M ± SD*)	Male RE (*n* = 343) (*M ± SD*)	Female RE (*n* = 298) (*M ± SD*)
Age (years)	28.8 ± 7.2	29.0 ± 5.7	28.5 ± 7.8	28.3 ± 6.7	29.4 ± 8.1
Height (cm)	176.8 ± 7.6	181.9 ± 3.9	168.5 ± 5.3	184.5 ± 5.8	168.1 ± 5.1
Weight (kg)	72.9 ± 15.2	92.4 ± 7.0	58.9 ± 6.7	84.1 ± 10.2	60.0 ± 8.2
BMI (kg/m^2^)	23.4 ± 3.4	27.5 ± 1.5	20.7 ± 1.9	23.8 ± 2.1	21.3 ± 2.8

Note. *N* = sample size; *M* = mean; *SD* = standard deviation. Height and weight values are self-reported. BMI was calculated as weight (kg)/height (m^2^). RTP = Resistance-Training Practitioners; RE = Recreational Exercisers. All somatometric indicators reflect self-reported data and should be interpreted accordingly.

**Table 2:** Descriptive Statistics by Subgroup.

Subgroup	*N*	Age (*M* ± *SD*)	Height (*M* ± *SD*)	Weight (*M* ± *SD*)	BMI (*M* ± *SD*)	Training Duration (*M* ± *SD*)	Training Frequency (*M* ± *SD*)
Male RTP	363	29.0 ± 5.7	182.0 ± 4.0	92.4 ± 7.0	27.5 ± 1.5	2.0 ± 0.2	5.23 ± 0.6
Female RTP	183	28.5 ± 7.8	168.5 ± 5.3	59.0 ± 6.7	20.7 ± 1.9	1.62 ± 0.5	3.64 ± 0.8
Male RE	343	28.3 ± 7.0	164.5 ± 5.9	84.1 ± 10.2	23.8 ± 2.1	1.62 ± 0.8	3.31 ± 0.7
Female RE	298	29.4 ± 8.1	168.1 ± 5.1	60.0 ± 8.2	21.3 ± 2.8	1.67 ± 0.8	3.35 ± 0.6

Note. *N* = sample size; *M* = mean; *SD* = standard deviation. Height (cm) and weight (kg) values are self-reported. BMI (kg/m^2^) was calculated as weight (kg) divided by height (m^2^). Training Duration refers to hours per session, and Training Frequency refers to sessions per week. RTP = Resistance-Training Practitioners; RE = Recreational Exercisers.

**Table 3:** Distribution of Sociodemographic and Behavioral Variables by Group (% of valid cases).

Variable	Categories	MRPT	FRPT	MRE	FRE
Marital Status	Free	84.8%	61.7%	74.1%	55.4%
	Married	15.2%	38.3%	25.1%	44.6%
	Divorced	–	–	0.9%	–
Education	Primary/Secondary	21.5%	19.1%	25.9%	8.7%
	University	69.7%	74.9%	47.2%	72.8%
	Master	8.8%	6.0%	26.8%	18.5%
Income (€)	0–500	2.5%	42.1%	13.7%	12.4%
	501–1000	57.0%	36.6%	47.2%	55.4%
	1001–1500	32.0%	17.5%	29.4%	25.8%
	1501–2000	8.0%	3.8%	6.4%	5.4%
Weight History	Yes	12.4%	6.0%	9.3%	0.0%
	No	87.6%	94.0%	90.7%	100.0%
Training Duration (hours)	1 h	1.7%	37.7%	54.5%	51.3%
	2 h	96.7%	62.3%	29.4%	30.2%
	3+ h	1.7%	–	16.0%	18.5%
Training Frequency (times/week)	3 times/week	–	36.1%	81.6%	70.8%
	4 times/week	8.0%	45.4%	9.0%	23.5%
	5 times/week	60.9%	12.6%	5.8%	5.7%
	6–7 times/week	31.2%	6.0%	3.5%	–
Double Training	Yes	5.5%	0.0%	2.6%	1.0%
	No	94.5%	100.0%	97.4%	99.0%
Sessions per Day	0	94.5%	100.0%	97.7%	100.0%
	1–2	5.5%	–	2.3%	–
Competition Participation	Yes	8.5%	0.0%	6.1%	3.4%
	No	91.5%	100.0%	93.9%	96.6%
Steroids Use	Never	30.3%	95.1%	95.3%	100.0%
	Rare	19.6%	4.9%	2.9%	–
	Occasional	22.0%	–	0.6%	–
	Frequent/Always	24.3%	–	1.2%	–
Laxatives Use	Never	67.2%	100.0%	98.3%	100.0%
	Rare/Occasional	32.8%	–	1.7%	–
Diuretics Use	Never	59.0%	98.9%	97.7%	100.0%
	Rare/Occasional	41.0%	–	2.3%	–

Note. M = Male; F = Female; RTP = Resistance-Training Practitioners; RE = Recreational Exercisers; MRPT = Male RTP; FRPT = Female RTP; MRE = Male RE; FRE = Female RE. Percentages represent valid percentages calculated after excluding missing values. The symbol “–” indicates 0% among valid cases.

**Table 4:** Mean Ranks for Training Frequency and Duration by Gender and Exercise Type.

Group	*N*	Training Frequency	Training Duration
Male RTP	363	965.5	741.0
Female RTP	183	527.0	541.6
Male RE	343	391.0	513.5
Female RE	298	416.5	539.7

Note. RTP = Resistance-Training Practitioners; RE = Recreational Exercisers. Mean ranks reflect the relative ordering of participants within each variable; higher ranks indicate greater training frequency or longer training duration.

**Table 5:** BMI and Weight-Related Difficulties by Income Group.

Income Group (€)	Mean BMI	% Reporting Weight Difficulties
0–500	26.9	18.2%
501–1000	24.9	12.4%
1001–1500	22.8	7.9%
1501–2000	21.9	5.6%
>2000	21.1	3.1%

Note. BMI values (kg/m^2^) represent group means. Weight-related difficulties refer to a self-reported history of problems with weight regulation or weight control.

**Table 6:** Binary Logistic Regression Predicting Exercise Type Based on Income and Education.

Predictor	B	*SE*	Wald	*p*-Value	Exp(B)
Income: €1001–1500	−0.599	0.311	3.722	0.054	0.549
Income: >€2000	1.345	0.795	2.861	0.091	3.839
Education: Postgraduate	1.330	0.238	31.253	<0.001 ***	3.782
Constant	−0.045	0.178	0.064	0.800	0.956

Note. Exercise type was coded as 1 = Resistance-Training Practitioners (RTP) and 0 = Recreational Exercisers (RE). Exp(B) represents the odds ratio. Statistically significant coefficients are shown in bold. *** *p* < 0.001.

**Table 7:** Substance Use by Exercise Type, Gender, and BMI.

Substance Category	Predominant Users	χ^2^ (df)	*p*-Value	BMI Association
Steroids	Male BB	344.7 (5)	<0.001 ***	Higher BMI (*p* < 0.001)
Laxatives	Occasional in Male BB	144.0 (3)	<0.001 ***	Not examined
Diuretics	Male BB	176.0 (3)	<0.001 ***	Not examined

Note. RTP = Resistance-Training Practitioners. χ^2^ values refer to Pearson Chi-Square tests. Statistically significant results are shown in bold. *** *p* < 0.001.

**Table 8:** Substance Use Frequency by Gender.

Substance Type	Frequency Category	Male (*n* = 706)	Female (*n* = 481)	Total (*N* = 1187)
Steroids	Never	437	472	909
	Rarely	81	9	90
	Sometimes	82	0	82
	Often	74	0	74
	Usually	18	0	18
	Always	14	0	14
Laxatives	Never	581	481	1062
	Rarely	103	0	103
	Sometimes	20	0	20
Diuretics	Never	549	479	1028
	Rarely	124	0	124
	Sometimes	30	0	30
	Often	2	0	2
	Usually	3	0	3

Note. Values represent absolute frequencies for each substance type and usage category.

**Table 9:** Logistic Regression Predicting Competition Participation.

Predictor	B	*SE*	Wald	*p*-Value	Exp(B)	95% *CI* for Exp(B)
Age	0.06	0.02	10.7	0.001 ***	1.06	[1.023, 1.096]
BMI	0.15	0.06	7.3	0.007 **	1.17	[1.043, 1.303]
Gender (Female)	−0.85	0.40	4.4	0.036 *	0.43	[0.194, 0.946]
Exercise Type (Recreational)	0.42	0.32	1.8	0.182	1.52	[0.821, 2.832]
Constant	−8.44	1.55	29.6	<0.001 ***	0.00	—

Note. The dependent variable is coded as 1 = participation, 0 = non-participation. Exp(B) indicates odds ratio; *CI* = confidence interval. Statistically significant results are highlighted in bold. * *p* < 0.05, ** *p* < 0.01, *** *p* < 0.001.

## Data Availability

The data presented in this study are available on request from the corresponding author due to privacy and ethical reasons.

## Abbreviations

The following abbreviations are used in this manuscript:

BMI	Body Mass Index
RTP	Resistance Training Practitioners
RE	Recreational Exercisers
MBB	Male Resistance Training Practitioners
FBB	Female Resistance Training Practitioners
MRE	Male Recreational Exercisers
FRE	Female Recreational Exercisers
MDI	Muscle Dysmorphia Inventory
